# Chronic intermittent hypoxia reshapes circadian metabolic architecture in a model of sleep apnea

**DOI:** 10.1126/sciadv.aeb3756

**Published:** 2026-02-25

**Authors:** Emilie Montellier, Guillaume Vial, Sophie Bouyon, Kousha Changizi Ashtiani, Sherif Abdelkarim, Emeline Lemarie, Antoine Boutin, Kenichiro Kinouchi, Pierre Baldi, Jean-Louis Pépin, Jonathan Gaucher

**Affiliations:** ^1^ Univ. Grenoble Alpes (UGA), Inserm (French National Institute of Health and Medical Research) Unit 1300, Hypoxia and Physiopathology Laboratory (HP2), Grenoble, France.; ^2^ Univ. Grenoble Alpes (UGA), Inserm (French National Institute of Health and Medical Research) Unit 1209, CNRS (French National Center for Scientific Research) Unit 5309, Institute for Advanced Biosciences (IAB), Grenoble, France.; ^3^ Department of Computer Science, University of California, Irvine, CA, USA.; ^4^ AI in Science Institute, University of California, Irvine, CA, USA.; ^5^ Division of Endocrinology, Metabolism, and Nephrology, Department of Internal Medicine, Keio University School of Medicine, Tokyo, Japan.; ^6^ Sleep Laboratory, Grenoble Alpes Hospital Center (CHUGA), Grenoble, France.

## Abstract

Obstructive sleep apnea (OSA), characterized by chronic intermittent hypoxia (IH) during sleep, is increasingly recognized as a driver of metabolic dysfunction. However, its impact on circadian metabolic regulation remains poorly understood. Here, we investigated how chronic IH reshapes 24-hour hepatic and systemic metabolic programs in a mouse model mimicking OSA-associated chronic hypoxia. Through integrated circadian transcriptomic, metabolomic, and physiological 24-hour profiling, we show that 4 weeks of rest phase–restricted IH reprograms hepatic and systemic metabolism in a time-specific manner. This reorganization involves the coordinated circadian regulation of glucose, lipid, and redox pathways, with a shift away from oxidative metabolism toward oxygen-sparing processes such as gluconeogenesis, glycogen turnover, and lipid mobilization. These changes align with the hypoxic phase exposure and coincide with reshaped hepatic metabolite oscillations and systemic autonomic rhythms, supporting a functional adaptation to intermittent oxygen availability. Mechanistically, we identify the cAMP-CREB1 pathway as a driver of circadian transcriptional remodeling in the liver and a central integrator of IH-dependent adrenergic stress. Our findings establish chronic IH as a potent metabolic zeitgeber that rewires hepatic transcriptional and metabolic programs, revealing a circadian dimension to the metabolic consequences of sleep-disordered breathing.

## Teaser

Sleep apnea reprograms circadian metabolism.

## INTRODUCTION

Circadian clock control of metabolism enables organisms to anticipate and adapt to environmental and physiological fluctuations (*1*–*4*). While the role of light, nutrient, and hormones in entraining circadian clocks is well established, hypoxia has only recently been recognized as a potential modulator of circadian rhythms (*5*–*13*). Diurnal variations in tissue oxygenation and oxidative metabolism have been documented across multiple organs, and short-term hypoxic exposure has been shown to elicit time-of-day–specific transcriptional responses in peripheral tissues (*6*, *11*, *12*, *14*, *15*). However, how chronic hypoxia affects circadian metabolic programs remains largely unknown.

Rodent models of obstructive sleep apnea (OSA) offer a unique opportunity to explore this question. OSA, the most prevalent sleep disorder, affects nearly one billion individuals globally (*16*). It is characterized by recurrent episodes of intermittent hypoxia (IH) during sleep, imposing a chronic, rest phase–restricted hypoxic stress tightly coupled to circadian physiology. Growing evidence suggests that chronic IH alone can drive metabolic dysfunction, including liver disease, in both clinical and experimental settings (*17*–*21*). The liver is a central hub of systemic metabolism and one of the most oxygen-demanding organs with tightly orchestrated 24-hour programs to regulate glucose, lipid, amino acid, cofactors, and xenobiotics metabolism (*22*–*25*). Hepatic metabolism is highly sensitive to both oxygen availability and sleep-wake cycle, two signals inherently intertwined in OSA. Given the central role of oxygen in energy metabolism and its chronic reduction during sleep in OSA, it is plausible that IH reshapes the liver’s temporal coordination of metabolic processes. IH is a very particular stimulus with the repetitive occurrence of desaturation-re-oxygenation sequences that can induce specific responses for both the clocks and metabolic system.

To investigate how chronic IH during sleep reshapes hepatic and systemic metabolism in a mouse model of OSA, we combined hepatic circadian transcriptomic and metabolomic profiling with continuous multidimensional physiological monitoring. Four weeks of IH induced a coordinated temporal reorganization of energy metabolism, marked by reinforcement of glycogen turnover and gluconeogenesis, and a dampening of tricarboxylic acid (TCA) cycle dynamics, aligning hepatic output with the exposure to time-restricted IH. This temporal reprogramming enhances hepatic glucose production and energy storage while limiting oxygen-dependent pathways. Mechanistically, we identify the cyclic adenosine monophosphate response element-binding protein 1 (CREB1) pathway as a driver of circadian transcriptional remodeling in the liver and a central integrator of IH-dependent adrenergic stress. These findings position chronic IH as a potent metabolic zeitgeber that entrains metabolism through time-specific transcriptional and physiological adaptations, unveiling a circadian dimension to metabolic alterations in sleep-disordered breathing.

## RESULTS

### Chronic IH reprograms circadian hepatic transcription

To dissect how chronic IH alters circadian metabolism, we first characterized longitudinal and circadian changes in systemic physiology in a mouse model of OSA. As previously described (*21*, *26*), mice progressively adjusted their circadian behavioral and physiological outputs over the course of IH exposure, with stabilization of body weight, food intake, glycemia, locomotor activity, and autonomic nervous system outputs by 3 to 4 weeks (fig. S1). These findings indicate that mice adapt to chronic IH through time-dependent physiological adjustments, suggesting a coordinated reprogramming of circadian metabolic regulation.

**Fig. 1.**
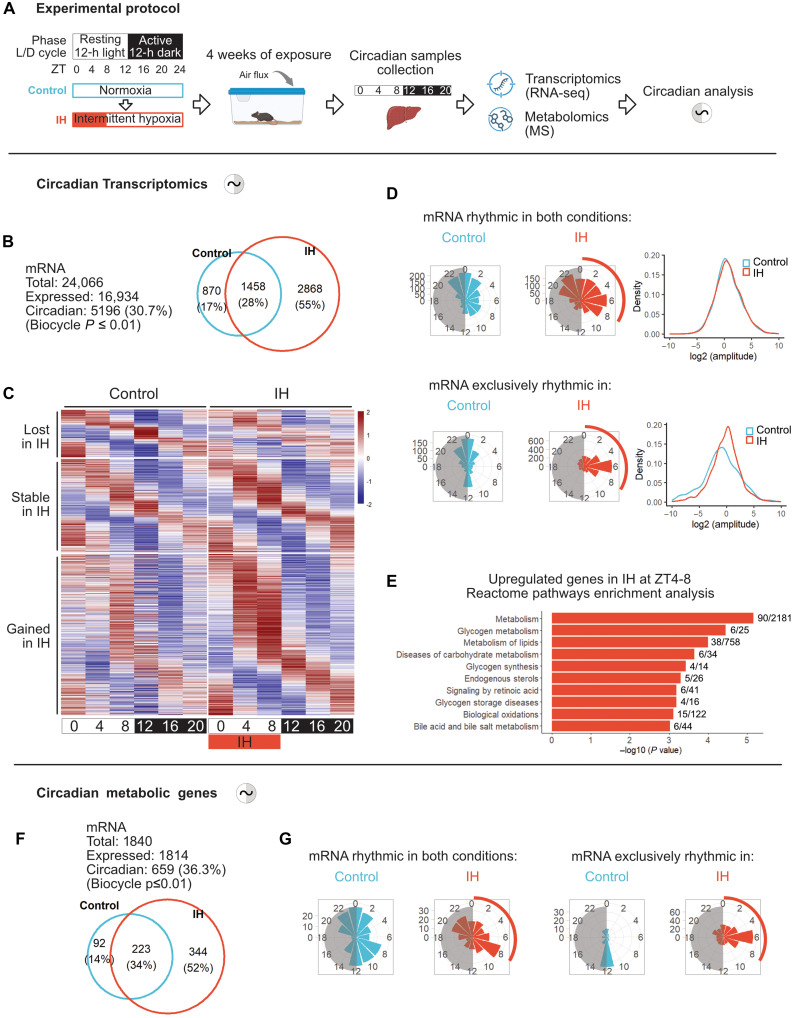
Chronic IH elicits a previously unknown circadian program of metabolic gene expression. (**A**) Experimental design. Mice were exposed to chronic intermittent hypoxia (IH) or normoxia (control) for 4 weeks. Liver samples were collected at six time points across the circadian cycle [Zeitgeber time (ZT); ZT0 = lights on] for circadian multiomics analyses. h, hour (**B**) Number of rhythmic genes in control and IH conditions identified using Biocycle (*P* ≤ 0.01, *n* = 3 animals per time point per condition). Venn diagram showing the number (top) and proportion (bottom) of rhythmic genes in each condition. (**C**) Heatmaps of phase-sorted expression for rhythmic transcripts identified exclusively in control (top, lost in IH; *n* = 870), exclusively in IH (bottom, gained in IH; *n* = 2868), or shared across both conditions (middle, stable in IH; *n* = 1458). (**D**) Phase and amplitude distributions for shared rhythmic genes (top; *n* = 1458) and condition-specific genes (bottom; control-specific *n* = 870, IH-specific *n* = 2868). Circular histograms show peak phases; density plots compare amplitude distributions. (**E**) Reactome pathway enrichment analysis of genes up-regulated at the main IH-specific expression peak (ZT6; differential analysis performed between ZT4 and ZT8; Cyber-T *P* ≤ 0.01, *n* = 3 animals per time point per condition). Numbers of genes in each pathway are shown to the right. (**F**) Number of rhythmic metabolic genes (gene set extracted from Reactome database) in control and IH (biocycle, *P* ≤ 0.01). Venn diagram displays number (top) and proportion (bottom) of rhythmic metabolic genes in each condition. (**G**) Phase analysis of rhythmic metabolic transcripts detected in both conditions (left, *n* = 223) or condition-specific (right; control, *n* = 92 and IH, *n* = 344). Circular histograms show peak phases. Created in BioRender. Vial, G. (2026) https://BioRender.com/ol2o32l.

To investigate the molecular basis of this adaptation, we applied a multiomics strategy in the liver, a central organ for systemic energy homeostasis and a key target of OSA-related metabolic dysfunction (Fig. 1A). We first analyzed how chronic IH reshapes the circadian organization of the hepatic transcriptome by sampling every 4 hours over a 24-hour cycle [Zeitgeber time (ZT); ZT0 = lights on] after 4 weeks of exposure. Rhythmic transcripts and their associated parameters (phase and amplitude) were determined using the BIO_CYCLE algorithm (*27*) and are accessible via the CircadiOmics web portal (*28*).

Of the 16,934 expressed transcripts in the liver, 5196 (30.7%) exhibited significant 24-hour rhythmicity in either condition. Notably, 2845 transcripts (55%) gained rhythmicity under IH, with a marked phase clustering around ZT6, coinciding with the hypoxic exposure, and exhibited increased amplitudes compared to control-specific oscillations (Fig. 1, B to D). In parallel, 1458 genes (28%) retained their rhythmicity under IH, with phases of expression broadly distributed over 12 hours and presenting a slight phase advance under IH. A relatively small subset of 870 genes (17%) lost rhythmicity under IH, primarily those peaking around ZT0 and ZT12, during light/dark transitions. This loss may reflect a selective dampening of transcriptional programs typically engaged at phase transitions, suggesting a temporal reprioritization under hypoxic stress. In contrast, the gain of rhythmicity and amplitude observed under IH highlights a coordinated adaptation that realigns hepatic functions with the recurring energetic constraints of the rest phase.

To gain insight into the functional consequences of hepatic circadian reprogramming under IH, we performed gene ontology (GO) enrichment analysis using curated gene sets from Reactome, BioPlanet, and the Molecular Signatures databases (fig. S2, A to C). Genes that gained rhythmicity under IH were enriched in pathways related to cell cycle regulation and growth factor signaling, consistent with transcriptional programs associated with liver injury and regeneration. In addition, transcripts up-regulated under IH with peaks near ZT6 were enriched for glycogen turnover and lipid metabolism, suggesting a temporally coordinated metabolic adaptation aligned with the hypoxic window (Fig. 1E). In contrast, genes that were rhythmic in control mice but lost rhythmicity under IH were associated with the TCA cycle and mitochondrial electron transport, pointing to a disruption of circadian oxidative metabolism. Genes that remained rhythmic across both conditions were enriched for core clock components and key metabolic functions, including cholesterol, fatty acid, and cofactor metabolism, highlighting the resilience of clock gene expression and the preservation of essential metabolic rhythms under hypoxic stress. Collectively, these findings support the concept that IH acts not only as a driver of hepatic stress but also as a metabolic modulator aligning liver gene expression with recurrent oxygen desaturation.

Building on these findings, we next focused our analyses on a curated set of metabolic genes (*n* = 1840) retrieved from the Reactome pathway database. Among the 1814 expressed metabolic genes in the liver, 659 (36.3%) exhibited significant 24-hour rhythmicity in at least one condition. IH induced a pronounced gain in their rhythmicity: 344 transcripts (52%) acquired circadian oscillations, 223 (34%) maintained them, and only 92 (14%) lost rhythmicity compared to controls (Fig. 1F and fig. S3A). Phase analysis showed that newly rhythmic metabolic transcripts peaked predominantly around ZT6, aligning with the hypoxic window (Fig. 1G). This temporal alignment mirrors the global transcriptomic shift and suggests that metabolic pathways are entrained as part of a broader coordinated circadian reprogramming rather than being independently rephased.

To further connect these transcriptional changes to metabolic outputs, we used the Human Metabolome Database (HMDB) to map rhythmic genes to their associated metabolites (fig. S3C). Metabolites predicted to oscillate only under IH included magnesium, l-serine, glycogen, and multiple triglyceride species, suggesting enhanced regulation of energy storage and lipid remodeling. In contrast, metabolites whose rhythmicity persisted in both conditions, such as NAD, choline, carbon dioxide, and pyrophosphate, reflect the maintenance and potential reinforcement of essential circadian metabolic outputs, suggesting that IH preserves tightly controlled pathways critical for hepatic homeostasis. Notably, metabolites predicted to be rhythmic only in controls [e.g., coenzyme Q (CoQ) species (QH2 and ubiquinone Q1) and succinyl-CoA] are central to mitochondrial oxidative phosphorylation and the TCA cycle, reinforcing our transcriptomic observation of a dampened circadian oxidative program under IH.

Together, these data highlight chronic IH as a strong metabolic zeitgeber that not only entrains hepatic gene expression to oxygen fluctuations but also shifts the balance from oxidative to oxygen-sparing metabolic pathways, reshaping the temporal organization of energy metabolism.

### Chronic IH drives selective circadian remodeling of hepatic metabolic rhythms

**Fig. 2.**
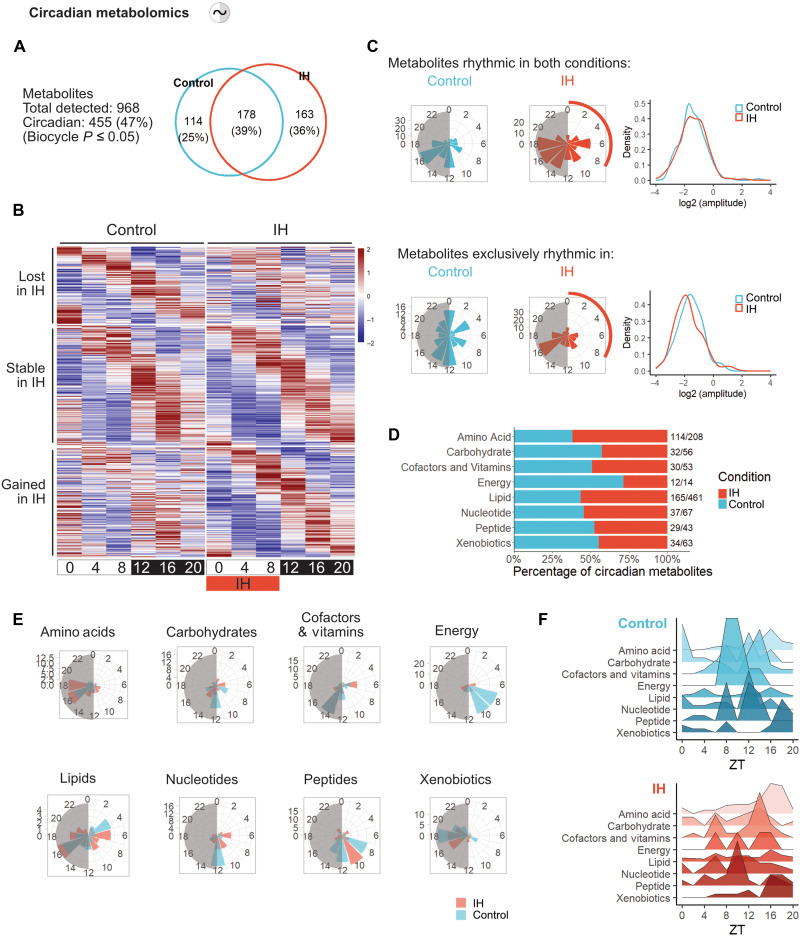
Chronic IH drives selective circadian remodeling of hepatic metabolic rhythms. (**A**) Number of rhythmic metabolites detected in control and IH livers (biocycle, *P* ≤ 0.05; *n* = 5 animals per time point per condition). Venn diagram indicates the number (top) and proportion (bottom) of rhythmic metabolites per group. (**B**) Phase-sorted heatmaps of rhythmic metabolites identified as control-specific (top; lost in IH, *n* = 114), IH-specific (bottom; gained, *n* = 163), or shared between both conditions (middle; stable, *n* = 178). (**C**) Phase and amplitude distributions of rhythmic metabolites. Top: shared metabolites (*n* = 178). Bottom: condition-specific metabolites (control: *n* = 114; IH: *n* = 163). Circular histograms show phase peaks; density plots display amplitude distributions. (**D**) Functional classification of rhythmic metabolites. Left: proportion of rhythmic metabolites per chemical class (amino acids, lipids, carbohydrates, etc.) for each condition. Right: absolute number of rhythmic metabolites relative to the total detected per class. (**E**) Peak phase distribution of rhythmic metabolites by chemical class in control and IH. Circular histograms illustrate phase clustering for each class. (**F**) Temporal landscape of rhythmic metabolites across the 24-hour cycle in control (top) and IH (bottom) livers.

To determine whether the transcriptional reprogramming of metabolic genes induced by IH translates into functional metabolic changes, we performed untargeted mass spectrometry (MS)–based metabolomics on the same liver samples collected every 4 hours across 24 hours (Fig. 1A). We detected 968 metabolites, nearly half of which (*n* = 455; 47%) exhibited significant circadian oscillations in either conditions. Among these, 163 metabolites (36%) gained rhythmicity under IH, 178 (39%) maintained their oscillations, and 114 (25%) lost rhythmicity (Fig. 2, A and B). This distribution suggests that while core metabolic rhythms remain largely intact, IH selectively remodels the temporal patterning of specific metabolic pathways. Metabolites gaining or maintaining rhythmicity under IH predominantly peaked around ZT16 during the active phase, whereas those losing rhythmicity clustered around ZT0 and ZT12 during light/dark transitions, paralleling the results observed at the transcriptional level (Fig. 2, B and C).

To further dissect this hepatic metabolic reprogramming, we analyzed rhythmic metabolites by functional class. IH enhanced circadian oscillations in amino acid (from 62 to 100 rhythmic metabolites) and lipid classes (from 92 to 119 lipid species), while rhythms in energy-related and carbohydrate metabolites were selectively reorganized (from 10 to 4 and from 27 to 20, respectively), with a similar pattern observed for cofactors and vitamins (25 to 24 rhythmic metabolites) (Fig. 2D). Temporal analyses revealed phase shifts in key metabolite classes: Energy metabolites lost their typical ZT8 to Z10 peak, and both carbohydrate and nucleotide metabolites exhibited disrupted ZT12 peaks, resulting in fragmented rhythms under IH (Fig. 2, E and F). In contrast, amino acids, lipids, cofactors, and vitamins retained coherent phasing, supporting the notion of a coordinated metabolic reallocation (Fig. 2, E and F). Notably, orotate, a metabolite tightly linked to feeding rhythms (*29*, *30*), maintained its circadian phase, indicating that the observed metabolic reprogramming likely reflects direct entrainment of hepatic metabolism by time-restricted hypoxia rather than behavioral changes (fig. S4A).

**Fig. 3.**
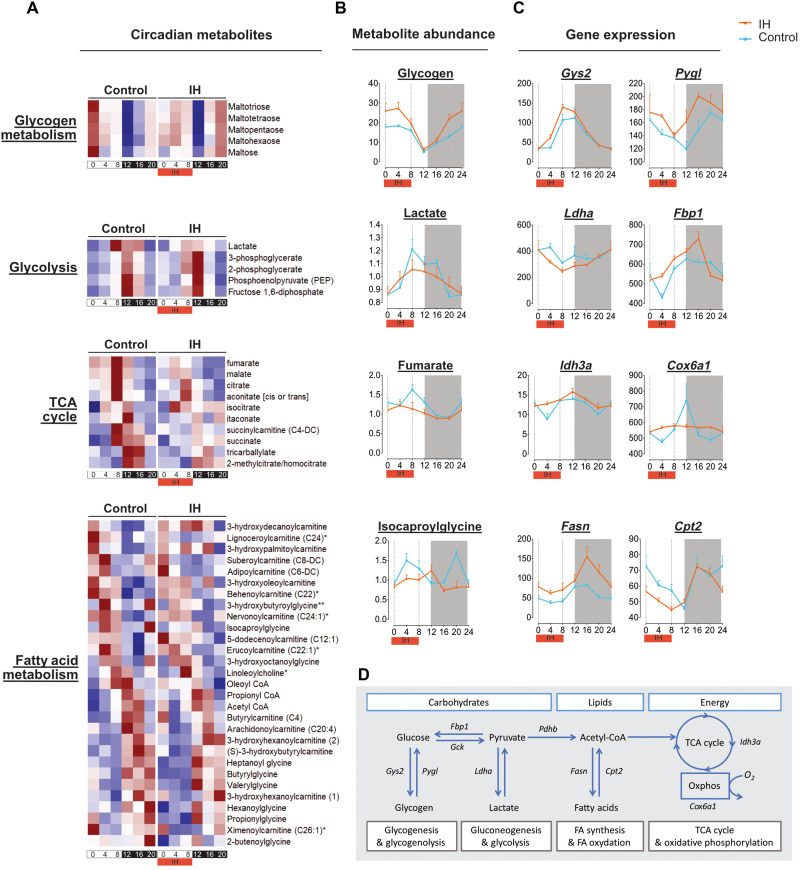
Chronic IH reprograms circadian regulation of hepatic metabolites and rate-limiting enzymes. (**A**) Phase-sorted heatmaps of rhythmic hepatic metabolites within central metabolic pathways under control or IH conditions, including glycogen metabolism, glycolysis/gluconeogenesis, the tricarboxylic acid (TCA) cycle, and fatty acid (acylcarnitine) profiles. Rhythmic metabolites were identified using biocycle analysis (*P* ≤ 0.05; *n* = 5 mice per time point per condition). [(B) and (C)] Temporal profiles of representative rhythmic metabolites (**B**) and their corresponding rate-limiting enzymes (**C**) across key metabolic pathways: glycogen turnover (*Gys2* and *Pygl*), glycolysis/gluconeogenesis and lactate metabolism (*Fbp1* and *Ldha*), TCA cycle and mitochondrial electron transport (*Idh3a* and *Cox6a1*), and fatty acid synthesis/oxidation (*Fasn* and *Cpt2*). Metabolic data: *n* = 5; transcriptomic data: *n* = 3 mice per time point per condition. Data are presented as mean ± SEM. (**D**) Schematic overview of rate-limiting enzymes within carbohydrate, lipid, and energy metabolism pathways that are temporally reorganized under chronic IH.

These alterations reflect a temporal reprioritization of key metabolic pathways, exemplified by the coordinated circadian transcriptional regulation of rate-limiting enzymes at critical metabolic branch points across lipid, carbohydrate, amino acid, and energy metabolism (Fig. 3, A to D).At the level of carbohydrate metabolism, core glycolytic and gluconeogenic enzymes, including *Gck* and *Fbp1*, retained robust rhythmicity under IH, in parallel with preserved oscillations in key glycolytic and gluconeogenic intermediates (Fig. 3C and fig. S4A). This suggests a maintained temporal coordination of glucose production and utilization. Both hepatic lactate levels and *Ldha* expression were dampened under chronic IH. While lactate typically accumulates in acute hypoxia, this observation may reflect a distinct adaptation to chronic IH, possibly involving altered glycolytic flux or systemic lactate clearance (Fig. 3, B and C). In parallel, genes involved in glycogen turnover, such as *Gys2* and *Pygl*, exhibited increased rhythmic expression under IH, accompanied by higher amplitude of hepatic glycogen oscillations (Fig. 3, A to C). This regulation likely reflects an anticipatory adaptation aimed at optimizing glycogen storage capacity and enabling rapid glucose mobilization in response to recurring hypoxic stress.

At the level of energy metabolism, chronic IH disrupted rhythmicity in key TCA cycle intermediates such as fumarate, citrate, and succinate, along with dampened expression of *Idh3a*, a rate-limiting TCA enzyme (Fig. 3, A to C, and fig. S4A). In parallel, *Cox6a1*, a subunit of cytochrome c oxidase (complex IV) involved in mitochondrial oxygen transfer, also lost rhythmic expression, pointing to a temporally constrained oxidative capacity (Fig. 3C). Together, these alterations reveal a circadian reorganization of mitochondrial metabolism, possibly restricting oxidative ATP production to cope with recurrent hypoxic stress.

In the context of lipid metabolism, rhythmic expression of *Fasn* and *Cpt2*, key enzymes in fatty acid synthesis and oxidation, respectively, was accompanied by de novo rhythmicity of mitochondrial byproducts of β-oxidation, such as isocaproylglycine and valerylglycine (Fig. 3, A to C, and fig. S4A). These changes point to a temporal reorganization of substrate utilization across the day-night cycle, potentially favoring lipid oxidation during hypoxic periods.

Regarding amino acid metabolism, oscillations were generally preserved under IH, with enhanced rhythmicity observed for several amino acids and related intermediates. These changes may reflect time-specific adjustments in nitrogen handling and amino acid catabolism, potentially influencing arginine-derived nitric oxide pathways and contributing to mitochondrial fueling under recurrent hypoxic stress.

Collectively, these adjustments converge on a broader reprogramming of mitochondrial energy metabolism, encompassing glucose mobilization, lipid oxidation, and TCA cycle activity. These findings position chronic IH as a potent metabolic zeitgeber that selectively reprograms the circadian architecture of hepatic metabolism, aligning energy allocation and substrate utilization with the recurring rest phase–restricted hypoxic stress.

### Chronic IH rewires circadian hepatic metabolism via CREB1 signaling

**Fig. 4.**
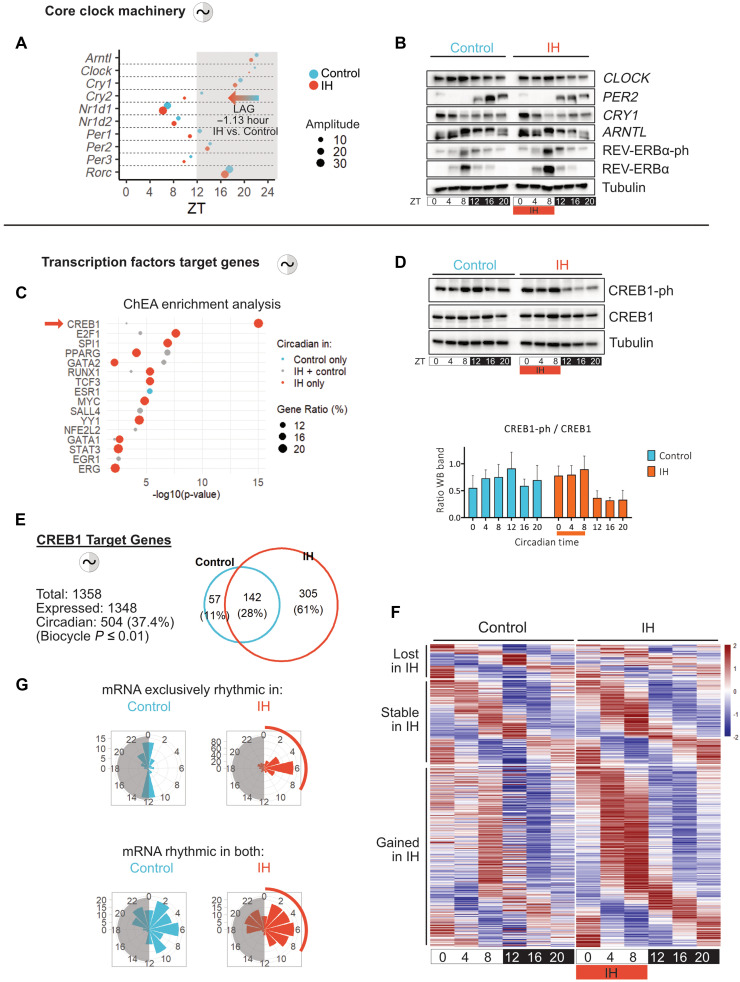
Chronic IH induces CREB1 activation and hepatic clock reorganization. (**A**) Phase and amplitude analysis of core clock gene expression in control and IH livers (RNA-seq; *n* = 3 animals per time point per condition). (**B**) Immunoblot analysis of core clock proteins in hepatic lysates from control and IH mice. Representative blots from three independent experiments using biological replicates; protein levels normalized to tubulin. (**C**) Transcription factor binding site (TFBS) enrichment of rhythmic genes detected exclusively in control, exclusively in IH, or in both conditions. Dot plot ranks TF motifs by *P* value and gene ratio. (**D**) Immunoblot analysis of total and phosphorylated CREB1 (CREB1-ph) in liver lysates. Representative blots from three independent experiments using biological replicates (upper panel); CREB1-ph and CREB1 protein levels were quantified and normalized to tubulin then the ratio of CREB1-ph/CREB1 was determined and plotted as mean ± SEM (lower panel). (**E**) Number and proportion of rhythmic CREB1 target genes in control and IH livers (biocycle, *P* ≤ 0.01; *n* = 3 mice per time point per condition). Venn diagram shows absolute numbers (top) and percentages (bottom). (**F**) Phase-sorted heatmaps of rhythmic CREB1 targets: Control-specific (top; lost in IH, *n* = 57), IH-specific (bottom; gained, *n* = 305), and shared (middle; stable, *n* = 142). (**G**) Circadian phase distribution of rhythmic CREB1 targets (RNA-seq; *n* = 3 animals per time point per condition). Circular histogram shows peak phases across the 24-hour cycle.

Given the extensive circadian reprogramming observed at both transcriptomic and metabolomic levels, we first examined whether core circadian oscillators remained functional under chronic IH. Core clock genes, including *Bmal1*, *Clock*, *Per*, *Cry* and *Rev-Erb* (*Nr1d*), *Ror* family members, maintained robust rhythmic expression under IH, with a modest global amplitude increase and a ~1-hour phase advance (Fig. 4A and fig. S4B). At the protein level, oscillations of core clock components BMAL1, CLOCK, PER2, CRY1, and REV-ERBα were also maintained, suggesting that the molecular clock machinery remains largely intact under IH despite a noticeable increase in amplitude and phosphorylation of REV-ERBα (Fig. 4B).

Thus, to identify complementary regulatory mechanisms, we performed transcription factor (TF) binding site (TFBS) enrichment analysis on the rhythmic gene sets. Among the top candidates, the cAMP response element-binding protein CREB1 emerged prominently (Fig. 4C). Known to integrate nutrient, hormonal, and stress cues, CREB1 is a central regulator of hepatic metabolism and is classically activated via phosphorylation through the cAMP–protein kinase A (PKA) pathway, notably in response to sympathetic stimuli, pathways plausibly engaged under IH (*15*, *31*–*35*). To test this, we assessed CREB1 protein expression and phosphorylation across the 24-hour cycle. Total CREB1 levels remained stable in both conditions (Fig. 4D). However, CREB1 phosphorylation exhibited distinct temporal dynamics: While a weak trend was observed in control animals (ZT8 to ZT12), a pronounced circadian peak emerged under IH between ZT0 and ZT8, aligning with the hypoxic phase (Fig. 4D). These changes suggest that IH promotes a rhythmic activation of CREB1 without altering its overall abundance. Supporting this, IH induced rhythmic expression in 305 of known CREB1 target genes (61%), with a pronounced peak around ZT6 (Fig. 4, E to G), closely matching the global rephasing of the IH transcriptome (Fig. 1). CREB1 targets included key rate-limiting enzymes involved in gluconeogenesis and glycogen turnover, such as *Pck1*, *G6pc*, *Fbp1*, and *Pygl*, which showed both increased expression and enhanced rhythmicity under IH (Fig. 3C and fig. S4A). This coordinated temporal activation supports a targeted up-regulation of glucose-producing pathways in response to chronic hypoxic stress.

**Fig. 5.**
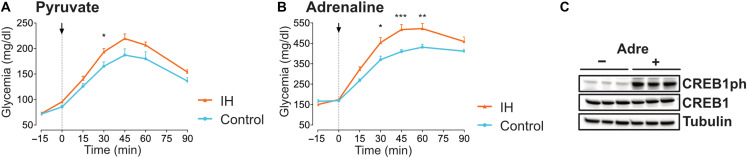
Chronic IH reorganizes hepatic gluconeogenic and adrenergic responses. (**A**) Pyruvate tolerance test (PTT) at ZT6 in 18-hour-fasted mice after 4 weeks of IH or control exposure (*n* = 6 animals per group). Blood glucose levels following intraperitoneal pyruvate (2 g/kg) and area under the curve (0 to 60 min) reflect hepatic gluconeogenic capacity. Data are presented as mean ± SEM. Multiple unpaired *t* test were performed at each time point with Šídák correction for multiple comparisons (*P < 0.05, **P < 0.01, ***P < 0.001, and ****P < 0.0001). (**B**) Glycemic response to intraperitoneal adrenaline (1 mg/kg) at ZT6 in 6-hour-fasted mice after 4 weeks of IH or control exposure (*n* = 6 animals per group). Data are presented as mean ± SEM. Multiple unpaired *t* test were performed at each time point with Šídák correction for multiple comparisons (*P < 0.05, **P < 0.01, ***P < 0.001, and ****P < 0.0001). (**C**) Immunoblot analysis of hepatic CREB1 and phosphorylated CREB1 (CREB1-ph) at ZT6 after a 6-hour fasting period, in the control group under basal conditions (NaCl injection) or following intraperitoneal adrenaline administration (Adre 2 mg/kg). Liver tissues were collected 15 minutes after injection. *n* = 3 animals per group.

To evaluate the functional impact of this transcriptional programming, we performed pyruvate tolerance tests during the rest phase (ZT6) and observed that IH-exposed mice exhibited significantly amplified glucose production compared to controls (Fig. 5A). These findings validate that chronic IH enhances hepatic gluconeogenic capacity in anticipation of increased energy demands during hypoxia. Given that CREB1 phosphorylation is classically induced by adrenergic signaling, we tested whether sympathetic activation could recapitulate this hepatic adaptation. Adrenaline injection at ZT6 amplified glycemic responses in IH mice (Fig. 5B) and triggered CREB1 phosphorylation (Fig. 5C), consistent with enhanced glycogen mobilization and/or adrenergic sensitivity. These results link sympathetic activation to CREB1-mediated metabolic reprogramming under chronic IH.

### Chronic IH reorganizes circadian systemic glycemic rhythms and sympathetic outflow

**Fig. 6.**
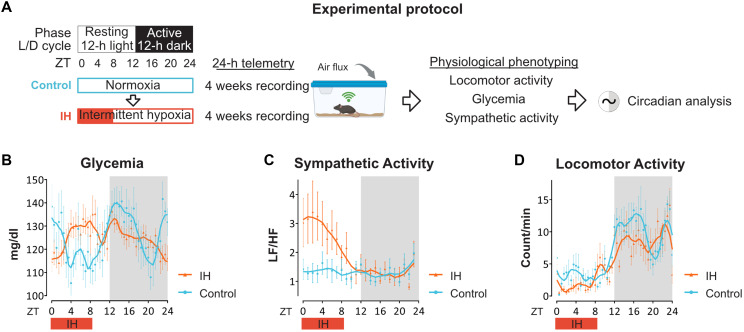
Chronic IH rewires circadian systemic glycemic rhythms and sympathetic outflow. (**A**) Experimental design for telemetric recordings. Mice were implanted with transmitters [ETA-F10 for locomotor/heart rate variability (HRV), *n* = 5 animals; HD-XG for continuous glucose *n* = 3 animals] and sequentially monitored under baseline normoxia (control) followed by 4 weeks of IH. (**B** to **D**) Circadian profiles under control (baseline) and intermittent hypoxia (IH). Longitudinal data were collected continuously and averaged over 30-min intervals for locomotor activity and glycemia, and 60-min intervals for HRV, then smoothed for graphical clarity. Group means ± SEM are shown (*n* = 5 for ETA-F10; *n* = 3 for HD-X10). (B) Continuous glycemia (HD-X10 sensor). Two-way repeated-measures analysis of variance (ANOVA) revealed a significant effect of time (*P < 0.05) and condition (****P < 0.0001), indicating circadian variation and a robust effect of IH. (C) Sympathetic tone [low-frequency to high-frequency (LF/HF) ratio, HRV analysis; ETA-F10 sensors]. Two-way repeated-measures ANOVA revealed a significant effect of time (**P < 0.01) and condition (****P < 0.0001), indicating circadian variation and a robust effect of IH. (D) Spontaneous locomotor activity (ETA-F10 sensors). Two-way repeated-measures ANOVA revealed a significant effect of time (****P < 0.0001) and condition (****P < 0.0001), indicating circadian variation and a robust effect of IH. Created in BioRender. Vial, G. (2026) https://BioRender.com/ol2o32l.

To assess whether the molecular and hepatic metabolic adaptations observed under IH parallel to changes in systemic physiology, we performed continuous telemetry monitoring of glycemia, heart rate variability (HRV), and locomotor activity across 24 hours (Fig. 6A). IH-exposed mice exhibited a marked redistribution of glycemic rhythms, characterized by a pronounced elevation in blood glucose levels during the light/rest phase, a period corresponding to the hypoxic window (Fig. 6B).

Notably, this hyperglycemic phase coincided temporally with a significant increase in the low-frequency to high-frequency (LF/HF) ratio, a proxy for sympathetic nervous system activity extracted from HRV analyses (Fig. 6C). This temporal alignment supports the idea that chronic IH entrains systemic glucose homeostasis via rhythmic sympathetic activation.

**Fig. 7.**
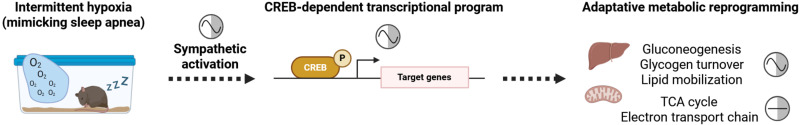
Proposed working model. Created in BioRender. Vial, G. (2026) https://BioRender.com/96q3cnm.

While glycemic and autonomic outputs were reprogrammed, locomotor activity and feeding rhythms remained largely preserved (Fig. 6D and fig. S1), indicating a selective uncoupling of feeding and locomotor behaviors and metabolic outputs under IH. This suggests that peripheral metabolic clocks, particularly in the liver, can adopt different temporal metabolic programs when driven by recurrent hypoxic and adrenergic cues (Fig. 7).

Together, these results support a model in which chronic IH imposes a distinct circadian architecture on cellular metabolism through time-specific activation of sympathetic signaling. This reprogramming strengthens the hepatic clock and CREB1-driven transcription, aligning energy allocation with the recurring metabolic demands imposed by chronic IH.

## DISCUSSION

By integrating circadian transcriptomic, metabolomic, and 24-hour physiological profiling, our study reveals that chronic IH, a hallmark of OSA, induces a time-of-day–specific reorganization of liver metabolic pathways, rather than a global desynchronization of the circadian system as previously described for acute, time-restricted, hypoxic stress (*9*, *15*). To capture the molecular determinants of this adaptation, we focused on a 4-week exposure to IH, a period during which physiological rhythms such as glycemia, food intake, locomotor activity, and sympathetic tone, stabilize after the initial fluctuations observed during short-term IH. This design enabled us to dissociate acute stress responses from long-term entrainment phenomena.

Notably, a large proportion of previously arrhythmic transcripts became rhythmic under IH, peaking predominantly around ZT6, during the hypoxic exposure window. IH also reinforces the rhythmicity of a large portion of genes strongly enriched in metabolic functions, including glycogen turnover, gluconeogenesis, lipogenesis, and sterol biosynthesis, with many encoding rate-limiting enzymes in these pathways such as *Pck1*, *Gys2*, *Cpt2*, *Fasn*, *Hmgcr*, or *Cyp7a1*. This coordinated transcriptional response was mirrored at the metabolite level, with noticeable circadian change in glycogen, lipids species, and TCA cycle intermediates. These findings support a model in which chronic IH acts as a temporal modulator of hepatic metabolism, selectively reinforcing the rhythmic coordination of pathways involved in energy mobilization and substrate allocation. Rather than broadly disrupting metabolic rhythms, IH reprograms their phase and amplitude to align key metabolic outputs with recurring physiological demands, thereby contributing to the maintenance of systemic homeostasis under sustained, time-restricted, hypoxic stress.

Chronic IH induces extensive de novo circadian rhythmicity with potential more limited contributions from acute hypoxic responses at the time of sampling. Several features indicate that the circadian remodeling we observe reflects a sustained adaptation rather than a transient stress response. The magnitude and coherence of the rhythmic transcriptional reprogramming induced by chronic IH far exceed what has been reported in acute hepatic hypoxia models (*9*, *15*), supporting the view that repeated hypoxic cycles drive long-term circadian entrainment. Longitudinal physiological and behavioral measurements throughout the 4-week exposure protocol further reveal progressive adjustments in arterial oxygen saturation, body weight, food intake, glycemia, locomotor activity, and HRV parameters. These gradual, time-dependent changes are consistent with chronic remodeling combined with acute reactivity and parallel clinical observations in sleep-disordered breathing, where brief sympathetic surges coexist with long-term alterations in autonomic regulation [see a reference review, (*36*)].

An important open question is whether the circadian reprogramming induced by chronic IH persists after cessation of hypoxic exposure and whether it depends on an intact molecular clock. While our current study focused on steady-state adaptations under chronic IH, future experiments could determine both the persistence and the clock-dependence of rhythmic metabolic and transcriptional responses, providing further insight into the mechanisms of long-term circadian remodeling.

A key finding of our study is the identification of CREB1 as a central effector linking sympathetic activation to liver circadian transcriptional reprogramming under chronic IH. While core clock components remained largely preserved, our data indicate that CREB1 target genes underwent extensive rhythmic remodeling, suggesting the existence of an autonomic-responsive circadian layer of transcriptional control. CREB1 is well known to integrate hormonal and stress-related cues, particularly through catecholamine-induced activation via the cAMP-PKA pathway during adrenergic stress (*31*–*33*). This upstream sympathetic input coincided with enhanced CREB1 phosphorylation at specific time points during the hypoxic window. Downstream, this activation enabled a time-specific amplification of key metabolic pathways, notably gluconeogenesis and glycogenolysis. Enzymes such as *Pck1*, *G6pc,* and *Pygl* showed increased expression and circadian amplitude under IH, in parallel with elevated hepatic glucose output in vivo. Together, these findings position CREB1 as a dynamic integrator of adrenergic signaling and circadian metabolic adaptation, coordinating input timing with output specificity in response to chronic hypoxic stress.

The CREB1-dependent transcriptional response observed in the liver under chronic IH emerges from a defined chromatin and metabolic landscape. While CREB1 activation is compatible with upstream catecholamine-driven cAMP-PKA signaling, additional modulation through hypoxia– or reactive oxygen species–dependent pathways is also plausible (*37*, *38*). The temporal profile of CREB1 phosphorylation likely reflects the integration of these convergent signals, enabling CREB1 to coordinate transcriptional outputs while remaining sensitive to feedback from the reshaped metabolic state. While this study focused on hepatic metabolism, comparable time-dependent CREB1 dynamics may occur in other organs exposed to IH, potentially integrating adrenergic and circadian cues to drive tissue-specific metabolic adaptation.

Acute and sustained hypoxia activates canonical HIF-dependent programs and can induce CREB signaling across multiple tissues (*9*, *38*). In contrast, chronic, rest-phase–restricted IH, modeling OSA, drives long-term adaptations across the 24-hour cycle, including a selective amplification of rhythmic CREB1 target genes in the liver, whereas classical HIF responses remain limited. The clock factor REV-ERBα is particularly relevant in this context. CREB1 and REV-ERBα act antagonistically at metabolic loci (*39*), and REV-ERBα, a heme-sensitive nuclear receptor (*40*, *41*), may be progressively modulated as hematocrit and heme rise under chronic IH. Future studies dissecting the temporal dynamics of HIF activation, heme accumulation, and CREB1–REV-ERBα interactions will be essential to test this working model.

The rhythmic nature of sympathetic activation itself appears to play a synchronizing role. The anticipated response to adrenaline suggests a metabolic and adrenergic remodeling upon chronic exposure to IH. Such coupling between autonomic cues and hepatic clocks provides a mechanistic basis for the selective alignment of glucose production and systemic homeostasis with periods of reduced oxygen availability. Telemetry recordings revealed that chronic IH reprograms 24-hour glycemia and sympathetic tone without altering locomotor activity, indicating that the autonomic response is driven by hypoxic exposure rather than behavioral changes. This supports the idea that rhythmic autonomic signaling can contribute to the entrainment of peripheral clocks independently of locomotor activity. It is important to note that locomotor activity, while commonly used as a proxy for rest-activity rhythms in rodent IH models, does not allow assessment of behavior and sleep architecture. Patients with OSA typically exhibit fragmented sleep and increased microarousals without major shifts in circadian sleep timing. Whether similar changes occur in chronic IH–exposed mice remains unknown, as this cannot be resolved without electroencephalography and electromyography recordings. Therefore, while our telemetry-based measurements demonstrate circadian reorganization of glycemia and sympathetic tone in the absence of overt changes in rest-activity patterns, future studies incorporating EEG-based sleep staging will be necessary to determine the extent to which alterations in sleep continuity contribute to these systemic adaptations.

From a translational perspective, our findings suggest that OSA-associated circadian reprogramming arises not only from IH per se but also from the autonomic responses it triggers, particularly recurrent adrenergic bursts that impose a pathological rhythmic input on peripheral clocks. Targeting circadian regulators such as CREB1, or modulating adrenergic signaling at specific times of day, may offer alternative strategies to restore temporal alignment and modulate metabolic output in patients with OSA.

Recent isotopic flux studies comparing acute and chronic sustained hypoxia have shown that adaptation to low oxygen involves organ-specific substrate selection. While acute hypoxia promotes glucose uptake and suppresses mitochondrial glucose oxidation across tissues, chronic hypoxia for 3 weeks enhances fatty acid metabolism and decrease glucose oxidation in the liver (*42*). These findings point to a shift in metabolic priorities that support long-term adaptation. Our results extend this framework by adding a temporal layer: Substrate usage in liver is not static but follows circadian patterns, with rhythmic glycolytic, gluconeogenic, and β-oxidation pathways. Our findings position chronic, time-restricted IH as a potent metabolic zeitgeber that selectively reprograms the circadian architecture of hepatic metabolism, aligning energy allocation and substrate utilization with the recurring time restricted hypoxic stress. Future studies using isotope tracers under circadian sampling will be instrumental to precisely quantify flux changes and assess how chronic IH entrains 24-hour metabolic programs across tissues.

Together, our work supports a model in which chronic IH acts as a metabolic zeitgeber, imposing previously unexplored temporal constraints on metabolically active organs and reshaping their circadian programs. Under normal conditions, hepatic clocks anticipate feeding and coordinate nutrient release with systemic demand. Under IH, this coordination is adaptively restructured: Peripheral clocks align with the recurring hypoxic cycles, synchronizing metabolic activity to fluctuations in oxygen availability and supporting organismal homeostasis.

## MATERIALS AND METHODS

### Animals and chronic IH exposure

Sixteen-week-old male C57BL/6JRj mice (Janvier Labs, Le Genest-Saint-Isle, France) were housed under a 12:12-hour light-dark cycle and maintained with ad libitum access to standard chow (LASQ Rodent Diet Rod16, LASVENDI) and water throughout the entire study, including the sampling day. To mimic the metabolic stress associated with OSA and associated IH, mice were exposed to a well-established chronic IH protocol consisting of 60 cycles per hour of alternating hypoxia [5% fraction of inspired oxygen (FiO_2_)] and room air (21% FiO_2_), restricted to the sleep/rest phase (ZT0 to ZT8). This chronic desaturation-reoxygenation paradigm reliably reduced mean arterial oxygen saturation to 60 to 70% during the first week of exposure and stabilize at ~75 to 80% after 2 weeks of chronic exposure (fig. S1A), reproducing the specific chronic hypoxic stress observed in patients with OSA as previously described (*21*, *43*–*46*). Control animals underwent matched air-air cycles to avoid bias from noise and turbulence related to gas flow. For circadian transcriptomic and metabolomic experiments, tissues were collected after 4 weeks of IH exposure, at 4-hour intervals across the 24-hour cycle, during either the active hypoxic phase or the subsequent normoxic periods. No fasting was performed before sampling to avoid introducing metabolic artifacts that could confound circadian analyses. At the indicated time points, mice were euthanized by decapitation, and tissues were rapidly excised and snap-frozen in liquid nitrogen for downstream analysis.

All experimental procedures were conducted in accordance with the European Convention for the Protection of Vertebrate Animals Used for Experimental and Other Scientific Purposes (ETS no. 123, Council of Europe, 1986) and the NIH Guide for the Care and Use of Laboratory Animals (NIH pub. no. 85-23, revised 1996). The study protocol was approved by the Institutional Animal Care and Use Committee under authorization number APAFIS no. 35029-2022012718108119 v6. Reporting of animal experiments adheres to the ARRIVE guidelines to ensure transparency and reproducibility.

### Food intake

Food intake was recorded at four time points evenly distributed over a 24-hour cycle, to assess circadian feeding patterns. At each time point, the feeder in each cage (housing five mice) was weighed, and the difference from the previous measurement was used to calculate food consumption over the preceding interval. Values were averaged per cage, providing group-level estimates of temporal feeding behavior. This protocol was repeated at each IH exposure week to monitor longitudinal adaptations.

### Telemetry recordings

Continuous glucose and cardiac telemetry recordings were acquired in freely moving mice under home-cage, ad libitum conditions during IH, using wireless HD-XG and ETA-F10 sensors [Data Sciences International (DSI)] as previously described (*26*). Devices were surgically implanted under isoflurane anesthesia (1.5 to 2% in a 50% air/50% oxygen mixture), and mice received pre- and postoperative analgesia with buprenorphine (0.05 to 0.1 mg/kg, subcutaneously). Animals were allowed a 2-week recovery period before data collection under control or chronic IH.

Glucose telemetry enabled high-resolution monitoring of glycemic fluctuations across the circadian cycle. HD-XG implants were calibrated in vivo by correlating telemetry signals with blood glucose measurements obtained via tail vein sampling using a glucometer (Accu-Chek) at multiple time points throughout the recording period. Cardiac function was assessed via electrocardiography (ECG) and HRV, with signal acquisition and analysis performed using the Ponemah software platform (DSI).

### Analysis of ECG recordings

Circadian HRVs were analyzed offline from 24-hour ECG recordings with labchart (LabChart v8, AD Instrument). The R wave of the QRS was detected automatically and the normal *R*-*R* intervals were determined. Ectopic beats, arrhythmias, and artifacts were removed using appropriate settings and Poincaré plot presentation in the HRV analysis window to search for outliers (*47*). The *R*-*R* values not contained between mean *R*-*R* interval ± 2 SD were excluded (*48*). Time and frequency domains were analyzed together over 12-hour light and 12-hour dark periods. From the 12 hours analyzed, the average of each parameter was calculated for each hour. Time-domain measures included mean *R*-*R* intervals (in ms), standard deviation of normal-to-normal intervals (SDNN in ms) and the root mean square of successive differences of normal-to-normal intervals (RMSSD in ms). For spectral analysis (frequency domain) of HRV, a power spectrum was obtained with a fast Fourier transform–based method. We defined the cutoff frequency range for low-frequency and high-frequency powers (LF, 0.15 to 1.5 Hz; HF, 1.5 to 5.0 Hz), we performed also the LF/HF ratio as a marker of sympathovagal balance.

### Oxygen saturation measurements

Oxygen saturation was noninvasively measured in mice using the MouseOx Plus pulse oximetry system (Starr Life Sciences Corp., Oakmont, PA, USA). Animals were lightly anesthetized with a low dose of ketamine/xylazine [e.g., ketamine (50 mg/kg) and xylazine (5 mg/kg), intraperitoneally] to minimize movement while preserving stable physiological conditions. Before measurements, mice were shaved at the neck area to ensure optimal sensor contact and signal quality. A neck collar sensor was carefully positioned around the shaved area to maintain consistent contact with the skin and reduce motion artifacts. Recordings were performed over a 7- to 10-min period once a stable signal was obtained.

### Metabolic tolerance tests

Adrenaline tolerance tests were performed after a 6-hour fast initiated at ZT0. At ZT5.5, mice were removed from the IH protocol and maintained in normoxia for a standardized 30-min recovery period before testing. Adrenaline (Renaudin; 1 mg/kg body weight) was administered intraperitoneally at ZT6, and blood glucose was measured from the tail vein using a glucometer (Accu-Chek) at 0 (baseline), 15, 30, 60, 90, and 120 min postinjection.

Pyruvate tolerance tests were performed after an 18-hour fast initiated at ZT12. At ZT5.5, mice were removed from the IH protocol and maintained in normoxia for a standardized 30-min recovery period before testing. Sodium pyruvate (Sigma-Aldrich, P2256; 2 g/kg body weight) was administered intraperitoneally at ZT6, and blood glucose was measured from the tail vein using a glucometer (Accu-Chek) at 0 (baseline), 15, 30, 60, 90, and 120 min postinjection.

Glucose tolerance tests were performed following a 6-hour fast initiated at ZT0. Mice received an intraperitoneal injection of glucose (2 g/kg body weight), and blood glucose levels were measured from the tail vein using a glucometer (Accu-Chek) at 0 (baseline), 15, 30, 60, 90, and 120 min postinjection.

### Glycogen extraction

Liver glycogen content was measured using the Glycogen Assay Kit (Abcam, ab65620) according to the manufacturer’s protocol, with minor modifications. Approximately 10 mg of liver tissue was homogenized on ice in 200 μl of ice-cold distilled water by vigorous vortexing. The homogenate was boiled for 5 min to inactivate endogenous enzymes, then transferred to a clean microcentrifuge tube. The original cryotube was rinsed with an additional 200 μl of distilled water to yield a final homogenate volume of 400 μl.

Samples were centrifuged at 13,000 rpm for 5 min at 4°C, and the supernatant was collected. A 1:20 dilution of the lysate was prepared (10 μl of lysate + 190 μl of distilled water). For each assay well, 25 μl of the diluted sample (or distilled water for the blank) was combined with 25 μl of hydrolysis buffer in a 96-well plate. After addition of 2 μl of hydrolysis enzyme mix, samples were incubated at room temperature for 30 min. Subsequently, 50 μl of reaction mix (46 μl of development buffer, 2 μl of development enzyme, and 2 μl of OxiRed probe) was added to each well and incubated for another 30 min at room temperature. Absorbance was measured at 570 nm using a microplate reader.

### RNA extraction and quantitative real-time PCR

Total RNA was extracted as previously described (*49*) from snap-frozen liver collected every 4 hours across 24 hours using TRIzol reagent, following the manufacturer’s instructions (Invitrogen, 15596026). Total RNA was isolated by precipitation with isopropanol and ethanol. Pellet was dissolved in diethyl pyrocarbonate–treated water (Invitrogen, 4622224). One microgram of RNA was reverse transcribed to cDNA using an iScript cDNA synthesis kit (Bio-Rad Laboratories, 1708840), according to the manufacturer’s protocol.

1/80e cDNA was used for quantitative real-time polymerase chain reaction (PCR) using SsoAdvanced SYBR Green Supermix (Bio-Rad Laboratories, 1725270). Gene expression was normalized to actin. Primer sequences used for gene expression analysis are listed in (*50*).

### RNA sequencing

Total RNA integrity was assessed using the Agilent TapeStation system with RNA ScreenTape, and purity was verified via absorbance ratios (260/280 nm and 260/230 nm). For mRNA sequencing (mRNA-seq), 1000 ng of total RNA in 25 μl was used as input for poly(A) selection using oligo(dT) magnetic beads. The library preparation was realized following manufacturer’s recommendations (stranded mRNA Prep, Ligation from ILLUMINA). The purified mRNA is fragmented and copied into first-strand cDNA using reverse transcriptase and random primers. In a second-strand cDNA synthesis step, deoxyuridine triphosphate replaces 3′-deoxythymidine 5′-triphosphate to achieve strand specificity. The final steps add adenine and thymine bases to fragment ends and ligate adapters. Libraries were amplified by PCR (10 to 11 cycles) and validated using TapeStation electrophoresis and Quantifluor quantification. All libraries were quantified, pooled in equimolar amounts, and sequenced on an Illumina NovaSeq 6000 platform using one S4 flow cell in XP mode, generating 2 × 100 bp paired-end reads. FastQ files were processed through the Tuxedo pipeline using TopHat and Cufflinks (*51*, *52*). RNA-seq reads were aligned to the mouse genome assembly (mm10) with TopHat using Bowtie2 as the aligner. Gene expression levels were quantified with Cufflinks, using a maximum bundle fragment threshold of 10,000,000. This pipeline yielded FPKM values for each gene in every replicate.

### Protein extraction and Western blotting

Liver protein was extracted as previously described (*50*). Fifty milligrams of liver was homogenized with Precellys (Bertin Technologies) at 6000 rpm, 3 × 10 s, 5-s pause, in modified RIPA buffer [50 mM tris-HCl (pH 8), 150 mM NaCl, 1% NP40, 0.5% sodium deoxycholate, 0.1% SDS, 0.5 μM trichostatin A, 10 mM nicotinamide, 20 mM NaF, 1 mM phenylmethylsulfonyl fluoride, and Complete protease inhibitor (Roche, 04693132001)], followed by a sonication at 20,000*g*, 10 min at 4°C.

Protein concentration was determined using Bradford Assay (Bio-Rad Laboratories, 5000006). Equal amounts of protein (generally 40 μg) were resolved by SDS–polyacrylamide gel electrophoresis on 4 to 20% polyacrylamide gels (Mini-PROTEAN, Bio-Rad, 4568096) and transferred to nitrocellulose membranes (Trans-Blot Turbo RTA Transfer Kit, Midi, Nitrocellulose, Bio-rad, 2201440). Membranes were blocked in 5% milk or 5% bovine serum albumin and incubated with primary antibodies overnight at 4°C, followed by horseradish peroxidase–conjugated secondary antibodies. Antibodies used for Western blots are as follows: anti-BMAL1 (Abcam, ab93806), anti-CLOCK (Cell Signaling Technology, 5157), anti-CREB (Cell Signaling Technology, 9197), anti-CREB–phospho-Ser^133^ (Cell Signaling Technology, 9198), anti-CRY1 (Bethyl Laboratories, A302-614), anti–REV-ERBa (Cell Signaling Technology, 13418), anti–REV-ERBα–phospho-Ser^55/59^ (Cell Signaling Technology, 2129), anti-PER2 (Alpha Diagnostic, PER21-A), anti-ACTIN (Sigma-Aldrich, A3853), anti–α-TUBULIN (Santa Cruz, sc5286), and anti-p84 (Genetex, GTX70220). For the secondary antibodies, goat–anti-mouse (Jackson ImmunoResearch, 115-035-003) and goat–anti-rabbit (Jackson Laboratories, 111-035-003) were used.

Detection was performed using chemiluminescence (Clarity Western ECL, Bio-Rad, 1705060) on a Bio-Rad ChemiDoc XRS+ imager. Relative protein levels were quantified by densitometry (Fiji software) and normalized to α-tubulin.

### Untargeted metabolomics

Untargeted metabolomics was performed on snap-frozen liver tissue harvested across six circadian time points using the services of Metabolon Inc. using a combination of ultrahigh-performance liquid chromatography-tandem MS (UPLC-MS/MS) platforms optimized for the detection of chemically diverse metabolites. Tissues were processed using the automated MicroLab STAR system. Proteins were precipitated using methanol under vigorous shaking to release small molecules and remove protein-bound metabolites. After centrifugation, the extracts were divided into aliquots for analysis under four chromatographic conditions: Two reverse-phase (RP) UPLC-MS/MS methods in positive ionization mode (optimized for early-eluting hydrophilic and late-eluting hydrophobic compounds), one RP-UPLC-MS/MS method in negative ion mode, and one hydrophilic interaction liquid chromatography–UPLC-MS/MS method in negative ion mode. Each method used a Waters ACQUITY UPLC system coupled to a Thermo Fisher Scientific Q-Exactive Orbitrap mass spectrometer with a heated electrospray ionization (HESI-II) source. The mass spectrometer alternated between full MS and data-dependent MSⁿ scans with dynamic exclusion, acquiring data at 35,000 resolutions over a mass-to-charge range of 70 to 1000. Raw data were processed using Metabolon’s proprietary informatics pipeline, which includes peak extraction, compound identification, quality control, and statistical normalization. Metabolites were identified by matching experimental features to an extensive internal library of over 5400 authentic standards and 7000 recurrent unknowns. Identification criteria included retention index, accurate mass (±10 parts per million), and MS/MS fragmentation patterns. Peaks were quantified by area under the curve. Data normalization included run-day correction to account for instrumental variability, ensuring comparability across all samples and time points.

### Circadian bioinformatics

Circadian analysis of time-series transcriptomic and metabolomic data was performed using BIO_CYCLE, a deep learning–based software developed to analyze periodicity in time-series data (*27*, *53*). To ensure robustness, rhythmicity detection was independently corroborated using the nonparametric JTK_Cycle method, enabling cross-validation of oscillatory features (*54*). For the final analysis, only transcripts with a BIO_CYCLE-derived *P* value ≤0.01 and metabolites with a *P* value ≤0.05 were considered significantly rhythmic and retained for downstream analyses. Heatmaps were generated using the R package pheatmap, Venn diagram were obtained using the R package Euleer, and phase/amplitude plots were constructed using the R package ggplot2.

Differential expression analysis between experimental conditions (e.g., normoxia control versus IH) was performed using Cyber-T, a Bayesian regularized *t* test designed for high-dimensional datasets (*55*, *56*). Significant differences were defined using a *P* value <0.01.

Gene set enrichment analysis (GSEA) was applied to assess functional enrichment of gene modules under different experimental conditions and time points (*57*). Gene sets with false discovery rate < 0.01 were considered significantly enriched.

Functional annotation and pathway analysis of oscillating and differentially expressed genes were performed using DAVID, the KEGG, and Reactome databases to identify enriched GO biological processes and canonical pathways (*58*–*60*).

Transcriptional regulation was investigated by identifying circadian genes and submitting them to Enrichr for TFBS enrichment analysis using the ChEA (ChIP-X Enrichment Analysis) database (*59*, *61*, *62*). This approach enabled the identification of TFs potentially regulating time-of-day–specific gene expression. In parallel, motif-based predictions of TF and RNA binding protein binding sites were performed using MotifMap and MotifMap-RNA, respectively (*63*, *64*).

Visualization and data exploration were facilitated using tools implemented in the CircadiOmics web platform (http://circadiomics.ics.uci.edu), where normalized expression and rhythm parameters (amplitude, phase, and period) were visualized across time points (*27*, *28*). Additional graphs and figures were generated using GraphPad Prism 10, R, and Affinity Designer.

### Statistics

We performed all statistical analyses using GraphPad Prism (version 9.5.1). Statistical significance was defined as *P* < 0.05. Data are presented as mean ± SEM. Figure legends detail the statistical test used for each experiment, and the *n* values reflecting the number of independent biological replicates. All analyses followed prespecified statistical procedures, with the sample size equal to the number of biological replicates (individual animals). No animals or data points were excluded.

Independent cohorts of mice were used for circadian transcriptomic/metabolomic profiling, telemetry recordings, and metabolic tests. This separation was necessary because surgical implantation, metabolic challenges, or repeated handling could alter circadian physiology and confound subsequent measurements. A summary of cohort allocation is provided in the fig. S5.

### Use of artificial intelligence

During the preparation of this manuscript, the authors used ChatGPT (GPT-4-class models, OpenAI, San Francisco, CA, USA) exclusively for refinement and correction purposes, including revising sentences to improve clarity, grammar, and scientific style while preserving the original meaning (e.g., prompt: “Could you revise this sentence to improve clarity, grammar, and scientific style while preserving the original meaning?”) and checking analysis code for clarity and consistency without altering the underlying computations (e.g., prompt: “Could you check this code for clarity and consistency, without altering the underlying computations?”). The authors reviewed and edited all content as needed and take full responsibility for the final version of the manuscript.
