# Supplementary material for: Chronic intermittent hypoxia reshapes circadian metabolic architecture in a model of sleep apnea
Source: Sci Adv. 2026 Feb 25;12(9):eaeb3756. doi: 10.1126/sciadv.aeb3756 (PMC12935056; doi:10.1126/sciadv.aeb3756)
Supplement: Supplementary file 1 — Figs. S1 to S5 [file sciadv.aeb3756_sm.pdf]

Science Advances
AAAS

Supplementary Materials for

Chronic intermittent hypoxia reshapes circadian metabolic architecture in a model of sleep apnea

Emilie Montellier *et al.*

Corresponding author: Jonathan Gaucher, jonathan.gaucher@univ-grenoble-alpes.fr;
Jean-Louis Pépin, jpepin@chu-grenoble.fr

Sci. Adv. **12**, eaeb3756 (2026)
DOI: 10.1126/sciadv.aeb3756

This PDF file includes:

Figs. S1 to S5

Fig. S1

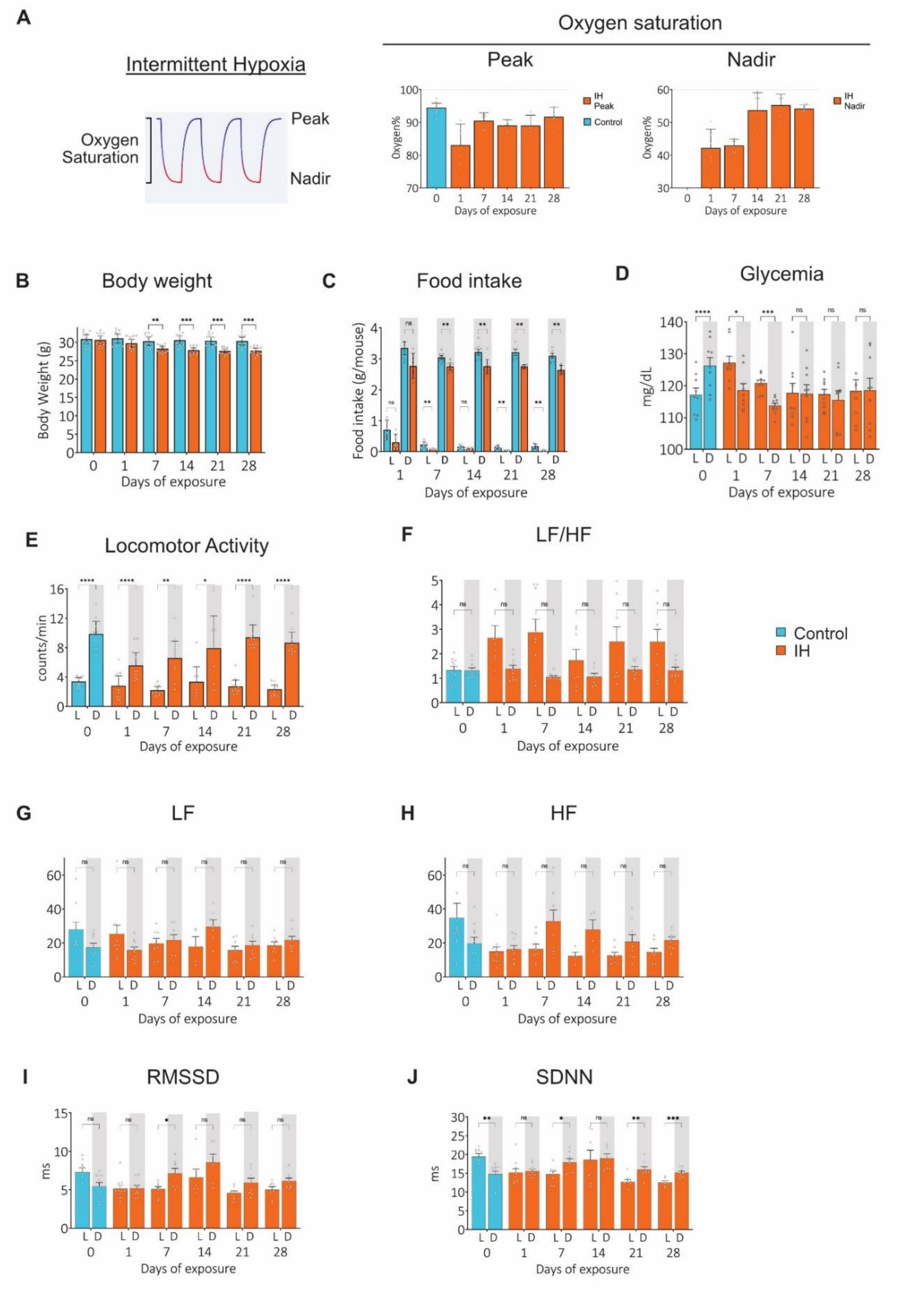
A
Oxygen saturation
Intermittent Hypoxia
Peak
Nadir
Oxygen Saturation
Peak
Nadir
Oxygen%
Days of exposure
IH Peak
Control
IH Nadir
B Body weight
Body Weight (g)
Days of exposure
C Food intake
Food intake (g/mouse)
Days of exposure
D Glycemia
mg/dL
Days of exposure
E Locomotor Activity
counts/min
Days of exposure
F LF/HF
Days of exposure
Control
IH
G LF
Days of exposure
H HF
Days of exposure
I RMSSD
ms
Days of exposure
J SDNN
ms
Days of exposure

Fig. S1. Time-course Physiological and Behavioral Monitoring during Chronic IH Exposure.

(**A**) Arterial oxygen saturation (SpO_2) measured weekly during daytime IH exposure (ZT6) across repeated hypoxia–reoxygenation cycles (30 s at 5% FiO_2 / 30 s at 21% FiO_2) over the 4-week protocol (days 1, 7, 14, 21, 28; n = 3). Values represent the mean nadir (desaturation) and peak (reoxygenation) SpO_2 across multiple cycles at each timepoint. Group means are presented with error bars representing ± SEM.

(**B**) Body weight measured weekly at ZT0 in IH (n = 15) and Control mice (n = 15) over the 4-week protocol (days 1, 7, 14, 21, 28). Data are presented as mean ± SEM. IH and Control groups were compared at each exposure time using unpaired t-test with Holm–Šídák correction for multiple comparisons (ns: not significant, *P < 0.05, **P < 0.01, ***P < 0.001, ****P < 0.0001).

(**C**) Food intake profiles across the light–dark cycle in IH (n = 15) and Control mice (n = 15) over the 4-week protocol (days 1, 7, 14, 21, 28). Data are presented as mean ± SEM. IH and Control groups were compared at each exposure time, both in light (L) and dark (D) phase, using unpaired t-test with Holm–Šídák correction for multiple comparisons (ns: not significant, *P < 0.05, **P < 0.01, ***P < 0.001, ****P < 0.0001).

(**D**) Glycemia profiles across the light–dark cycle obtained by continuous telemetry (n = 3 animals) over the 4-week protocol (days 1, 7, 14, 21, 28). Data are presented as mean ± SEM. Values of light (L) and dark (D) phase were compared at normoxia (Control) and at each exposure time of IH, using paired t-test with Holm–Šídák correction for multiple comparisons (ns: not significant, *P < 0.05, **P < 0.01, ***P < 0.001, ****P < 0.0001).

(**E**) Ambulatory locomotor activity profiles across the light–dark cycle (n = 5 animals) over the 4-week protocol (days 1, 7, 14, 21, 28). Data are presented as mean ± SEM. Values of light (L) and dark (D) phase were compared at normoxia (Control) and at each exposure time of IH, using paired t-test with Holm–Šídák correction for multiple comparisons (ns: not significant, *P < 0.05, **P < 0.01, ***P < 0.001, ****P < 0.0001).

(**F-J**) Heart rate variability (HRV) metrics derived from ECG telemetry (n = 5 animals) over the 4-week protocol (days 1, 7, 14, 21, 28): (**F**) LF/HF ratio, (**G**) LF power, (**H**) HF power, (**I**) RMSSD, (**J**) SDNN. Data are presented as mean ± SEM. Values of light (L) and dark (D) phase were compared at normoxia (Control) and at each exposure time of IH, using paired t-test with Holm–Šídák correction for multiple comparisons (ns: not significant, *P < 0.05, **P < 0.01, ***P < 0.001, ****P < 0.0001).

Fig. S2

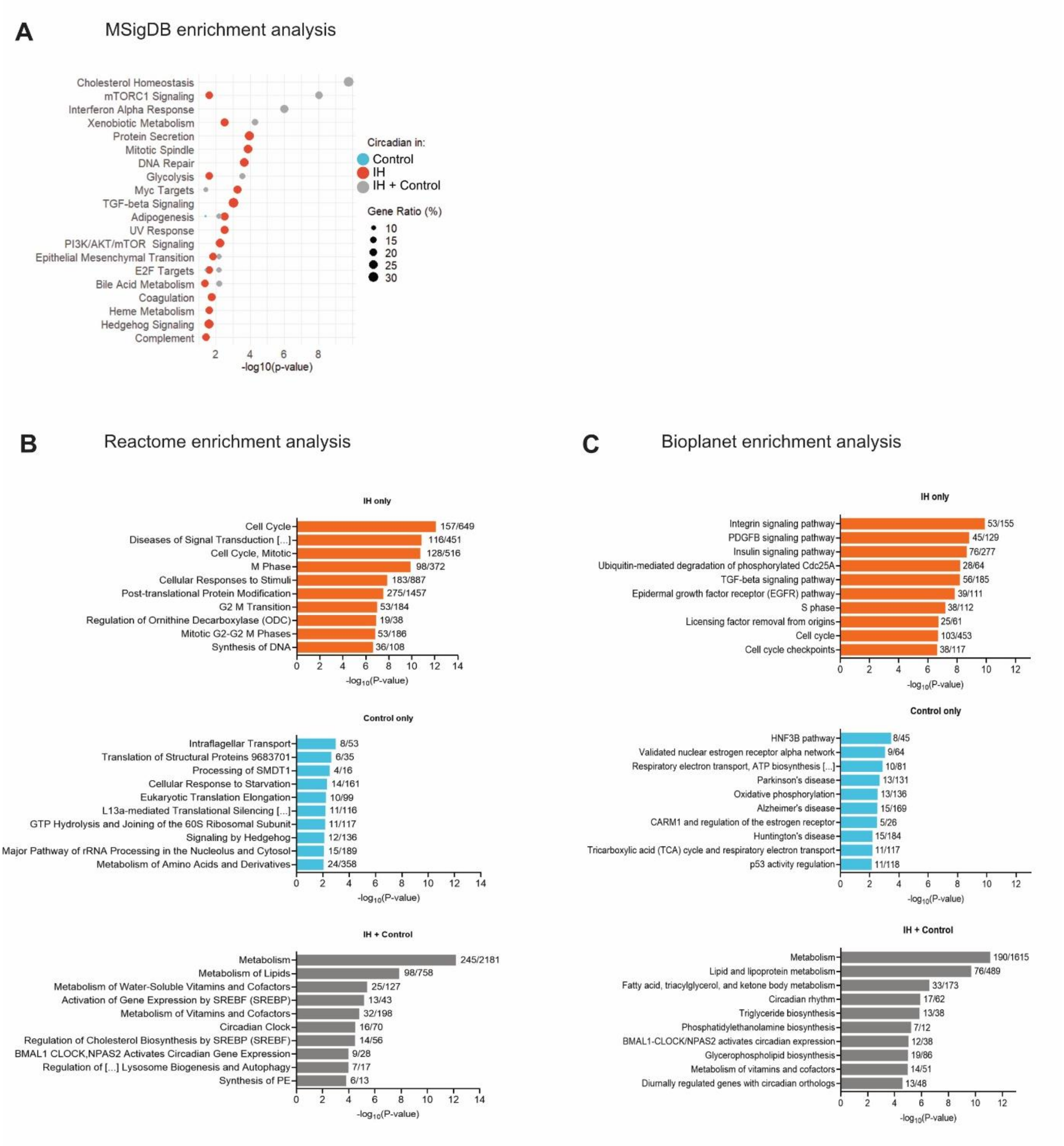
A
MSigDB enrichment analysis
Cholesterol Homeostasis
mTORC1 Signaling
Interferon Alpha Response
Xenobiotic Metabolism
Protein Secretion
Mitotic Spindle
DNA Repair
Glycolysis
Myc Targets
TGF-beta Signaling
Adipogenesis
UV Response
PI3K/AKT/mTOR Signaling
Epithelial Mesenchymal Transition
E2F Targets
Bile Acid Metabolism
Coagulation
Heme Metabolism
Hedgehog Signaling
Complement
-log10(p-value)
Circadian in:
Control
IH
IH + Control
Gene Ratio (%)
B
Reactome enrichment analysis
IH only
Cell Cycle 157/649
Diseases of Signal Transduction [...] 116/451
Cell Cycle, Mitotic 128/516
M Phase 98/372
Cellular Responses to Stimuli 183/887
Post-translational Protein Modification 275/1457
G2 M Transition 53/184
Regulation of Ornithine Decarboxylase (ODC) 19/38
Mitotic G2-G2 M Phases 53/186
Synthesis of DNA 36/108
Control only
Intraflagellar Transport 8/53
Translation of Structural Proteins 9683701 6/35
Processing of SMDT1 4/16
Cellular Response to Starvation 14/161
Eukaryotic Translation Elongation 10/99
L13a-mediated Translational Silencing [...] 11/116
GTP Hydrolysis and Joining of the 60S Ribosomal Subunit 11/117
Signaling by Hedgehog 12/136
Major Pathway of rRNA Processing in the Nucleolus and Cytosol 15/189
Metabolism of Amino Acids and Derivatives 24/358
IH + Control
Metabolism 245/2181
Metabolism of Lipids 98/758
Metabolism of Water-Soluble Vitamins and Cofactors 25/127
Activation of Gene Expression by SREBF (SREBP) 13/43
Metabolism of Vitamins and Cofactors 32/198
Circadian Clock 16/70
Regulation of Cholesterol Biosynthesis by SREBP (SREBF) 14/56
BMAL1 CLOCK,NPAS2 Activates Circadian Gene Expression 9/28
Regulation of [...] Lysosome Biogenesis and Autophagy 7/17
Synthesis of PE 6/13
$-log_{10}$(P-value)
C
Bioplanet enrichment analysis
IH only
Integrin signaling pathway 53/155
PDGFB signaling pathway 45/129
Insulin signaling pathway 76/277
Ubiquitin-mediated degradation of phosphorylated Cdc25A 28/64
TGF-beta signaling pathway 56/185
Epidermal growth factor receptor (EGFR) pathway 39/111
S phase 38/112
Licensing factor removal from origins 25/61
Cell cycle 103/453
Cell cycle checkpoints 38/117
Control only
HNF3B pathway 8/45
Validated nuclear estrogen receptor alpha network 9/64
Respiratory electron transport, ATP biosynthesis [...] 10/81
Parkinson's disease 13/131
Oxidative phosphorylation 13/136
Alzheimer's disease 15/169
CARM1 and regulation of the estrogen receptor 5/26
Huntington's disease 15/184
Tricarboxylic acid (TCA) cycle and respiratory electron transport 11/117
p53 activity regulation 11/118
IH + Control
Metabolism 190/1615
Lipid and lipoprotein metabolism 76/489
Fatty acid, triacylglycerol, and ketone body metabolism 33/173
Circadian rhythm 17/62
Triglyceride biosynthesis 13/38
Phosphatidylethanolamine biosynthesis 7/12
BMAL1-CLOCK/NPAS2 activates circadian expression 12/38
Glycerophospholipid biosynthesis 19/86
Metabolism of vitamins and cofactors 14/51
Diurnally regulated genes with circadian orthologs 13/48
$-log_{10}$(P-value)

Fig. S2. Gene Ontology (GO) Enrichment Analyses of Rhythmic Hepatic Transcripts under Control and IH Conditions.

(**A**) MSigDB enrichment analyses were performed on rhythmic genes detected exclusively in Control (lost under IH; N = 870), exclusively in IH (gained; N = 2,868), or shared across both conditions (stable; N = 1,458). Represented as a dot plot, ranking pathways by p-value (x-axis) with gene ratio indicated by dot size.

(**B**) Reactome enrichment analyses were performed on rhythmic genes detected exclusively in Control (lost under IH; N = 870), exclusively in IH (gained; N = 2,868), or shared across both conditions (stable; N = 1,458). Represented as bar graphs; pathways are ranked by p-value, with gene ratio indicated on the right.

(**C**) BioPlanet enrichment analyses were performed on rhythmic genes detected exclusively in Control (lost under IH; N = 870), exclusively in IH (gained; N = 2,868), or shared across both conditions (stable; N = 1,458). Represented as bar graphs; pathways are ranked by p-value, with gene ratio indicated on the right.

Fig. S3

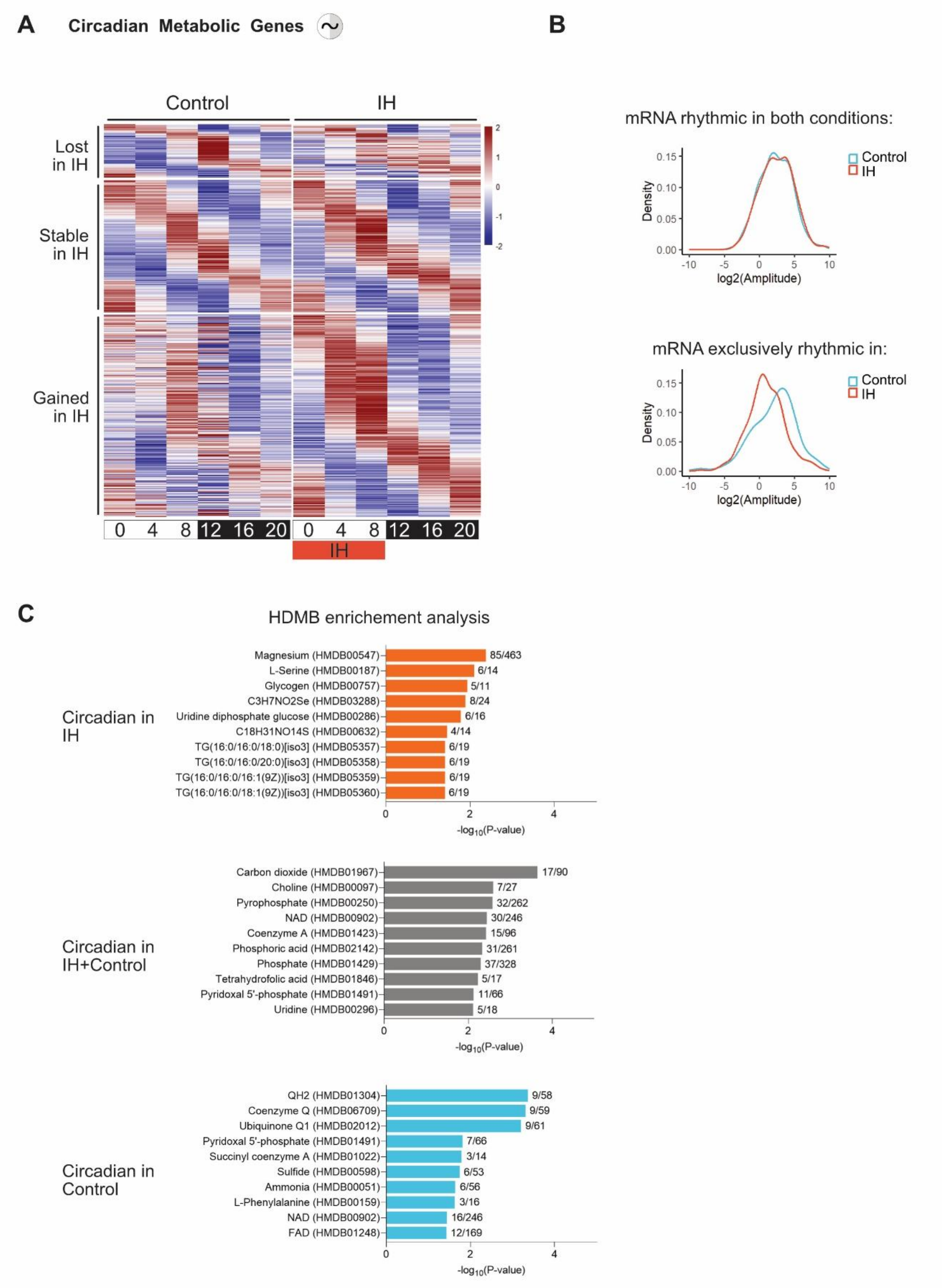
A
Circadian Metabolic Genes
Control
IH
Lost in IH
Stable in IH
Gained in IH
0 4 8 12 16 20 0 4 8 12 16 20
IH
B
mRNA rhythmic in both conditions:
Control
IH
Density
log2(Amplitude)
mRNA exclusively rhythmic in:
C
HDMB enrichement analysis
Circadian in IH
Magnesium (HMDB00547) 85/463
L-Serine (HMDB00187) 6/14
Glycogen (HMDB00757) 5/11
C3H7NO2Se (HMDB03288) 8/24
Uridine diphosphate glucose (HMDB00286) 6/16
C18H31NO14S (HMDB00632) 4/14
TG(16:0/16:0/18:0)[iso3] (HMDB05357) 6/19
TG(16:0/16:0/20:0)[iso3] (HMDB05358) 6/19
TG(16:0/16:0/16:1(9Z))[iso3] (HMDB05359) 6/19
TG(16:0/16:0/18:1(9Z))[iso3] (HMDB05360) 6/19
-log10(P-value)
Circadian in IH+Control
Carbon dioxide (HMDB01967) 17/90
Choline (HMDB00097) 7/27
Pyrophosphate (HMDB00250) 32/262
NAD (HMDB00902) 30/246
Coenzyme A (HMDB01423) 15/96
Phosphoric acid (HMDB02142) 31/261
Phosphate (HMDB01429) 37/328
Tetrahydrofolic acid (HMDB01846) 5/17
Pyridoxal 5'-phosphate (HMDB01491) 11/66
Uridine (HMDB00296) 5/18
Circadian in Control
QH2 (HMDB01304) 9/58
Coenzyme Q (HMDB06709) 9/59
Ubiquinone Q1 (HMDB02012) 9/61
Pyridoxal 5'-phosphate (HMDB01491) 7/66
Succinyl coenzyme A (HMDB01022) 3/14
Sulfide (HMDB00598) 6/53
Ammonia (HMDB00051) 6/56
L-Phenylalanine (HMDB00159) 3/16
NAD (HMDB00902) 16/246
FAD (HMDB01248) 12/169

Fig. S3. Circadian Reprogramming of Metabolic Gene Expression and Associated Metabolite Annotations under IH.

(**A**) Heatmaps showing phase-sorted expression profiles of rhythmic metabolic transcripts identified exclusively in Control (top; lost under IH, N = 870), exclusively in IH (bottom; gained under IH, N = 344), or shared across both conditions (middle; stable, N = 92).

(**B**) Amplitude distributions of rhythmic metabolic genes. Density plots show amplitude distributions for genes rhythmic in both conditions (top; N = 92) and those specific to each condition (bottom; Control-specific: N = 870; IH-specific: N = 344).

(**C**) Metabolite set enrichment analysis based on HMDB annotations. Bar graphs display metabolite associated with each rhythmic gene set (Control-only, IH-only, shared), ranked by p-value; gene-to-metabolite association ratios are indicated on the right.

Fig. S4

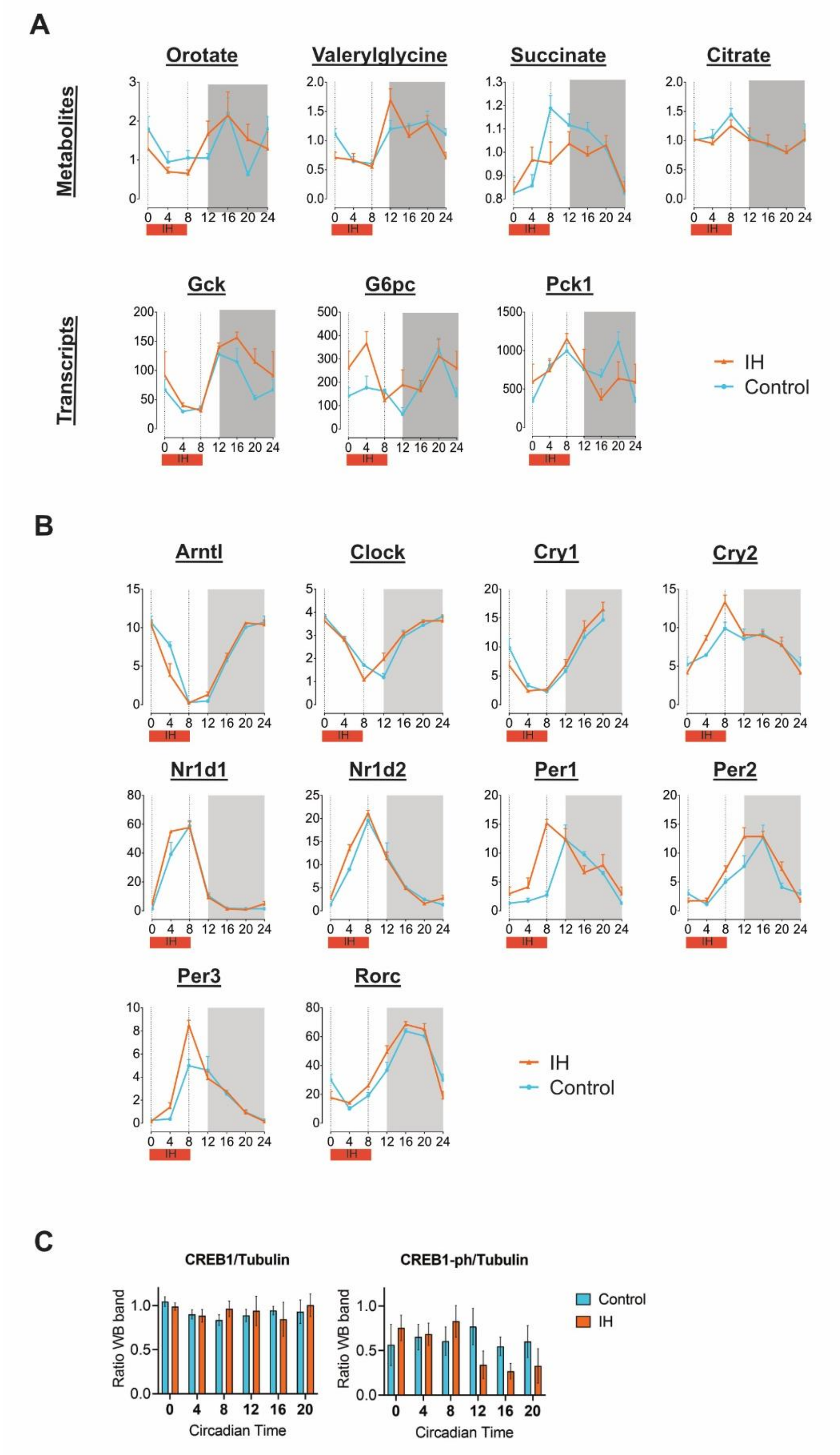
A
Metabolites
Orotate
Valerylglycine
Succinate
Citrate
Transcripts
Gck
G6pc
Pck1
IH
Control
B
Arntl
Clock
Cry1
Cry2
Nr1d1
Nr1d2
Per1
Per2
Per3
Rorc
IH
Control
C
CREB1/Tubulin
CREB1-ph/Tubulin
Ratio WB band
Circadian Time
Control
IH

Fig. S4. Circadian Profiles of Representative Metabolites and Core Clock Genes under Control and IH Conditions.

(**A**) Temporal profiles of selected rhythmic metabolites (top) and transcripts (bottom) across the 24-h cycle. Metabolite quantification: n = 5 mice per time point per condition; transcriptomic data: n = 3 mice per time point per condition. Data are presented as mean ± SEM.

(**B**) Circadian expression profiles of core clock genes measured by qPCR (n = 5 mice per time point per condition). Data are presented as mean ± SEM.

(**C**) Quantification of total CREB1 and phosphorylated CREB1 (CREB1-ph) protein levels in liver lysates. CREB1 and CREB1-ph intensities were quantified by densitometry and normalized to tubulin (n = 3 mice per time point per condition).

Fig. S5

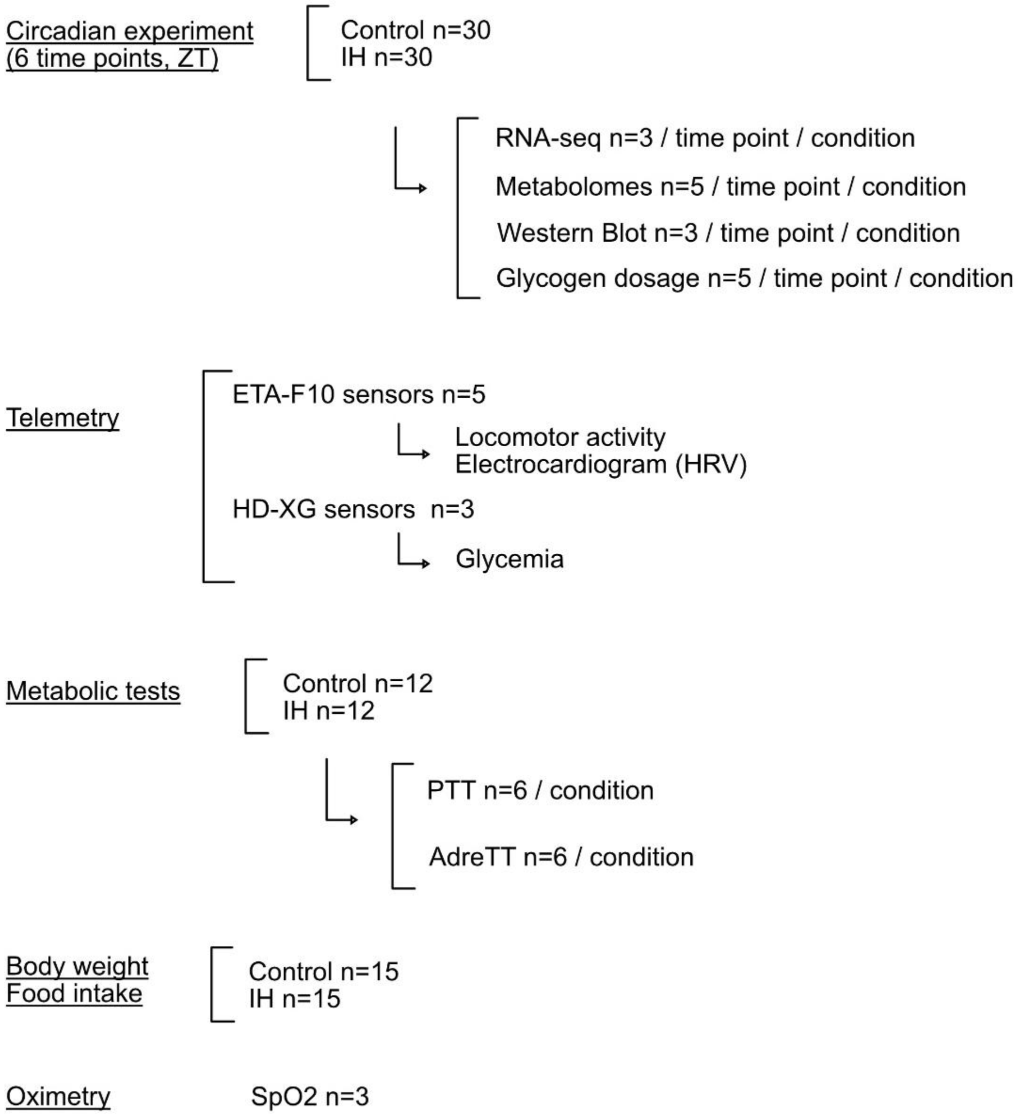
Circadian experiment
(6 time points, ZT)
Control n=30
IH n=30
RNA-seq n=3 / time point / condition
Metabolomes n=5 / time point / condition
Western Blot n=3 / time point / condition
Glycogen dosage n=5 / time point / condition
Telemetry
ETA-F10 sensors n=5
Locomotor activity
Electrocardiogram (HRV)
HD-XG sensors n=3
Glycemia
Metabolic tests
Control n=12
IH n=12
PTT n=6 / condition
AdreTT n=6 / condition
Body weight
Food intake
Control n=15
IH n=15
Oximetry
SpO2 n=3

Fig. S5. Schematic of the Experimental Design and Number of Animals Used in Each Experimental Set.
